# Health information-seeking behavior in patients with coronary artery disease: Activating methods

**DOI:** 10.1371/journal.pone.0300755

**Published:** 2024-04-17

**Authors:** Min-Song Kim, Sang-Hee Kim

**Affiliations:** 1 Yeungnam Medical Center, Daegu, Republic of Korea; 2 College of Nursing, Keimyung University, Daegu, Republic of Korea; An-najah National University Faculty of Medicine and Health Sciences, STATE OF PALESTINE

## Abstract

**Introduction:**

Coronary artery disease (CAD) has a high mortality rate worldwide, and continuous health behavior practice and careful management are required owing to risks such as rapid changes in symptoms and emergency hospitalization. The utilization of health-related information is an important factor for long-term disease management in patients with CAD. For this purpose, an understanding of health information-seeking behavior is needed first.

**Methods:**

This study analyzed data from the 2021 Korea Medical Panel Survey, and logistic regression analysis was conducted to confirm the factors influencing the health information-seeking behavior of patients with CAD.

**Results:**

The health information-seeking behavior of patients with CAD differed according to demographic characteristics, and differences in preferred information use were confirmed. Finally, it was identified that insufficient levels of health literacy were a major reason for CAD patients not engaging in health information-seeking behaviors (OR, 0.17; 95% CI, 0.09–0.33; p < 0.001).

**Conclusion:**

This study suggests that to improve health information-seeking behaviors, the application of education and intervention programs to increase the level of health literacy is necessary.

## Introduction

Cardiovascular disease ranks first among the world’s top 10 causes of death [1]. Chronic diseases require medication management and the improvement of healthy habits throughout life after an emergence, and long-term healthcare and preventive activities are essential. Coronary artery disease (CAD), which accounts for the largest proportion of heart diseases, requires careful health management because of its high risk of recurrence (even after acute treatment), rapid changes in symptoms, and high risk of emergency hospitalization [2, 3]. In particular, the absence of health-related behavior practices of CAD patients affect not only the deterioration and recurrence of the disease, but also readmission and associated mortality [4].

An important factor for a patient’s continuous health management is exploring and utilizing health information on their own [5]. This helps healthcare by improving patients’ self-management skills and understanding of health information to facilitate communication with medical staff [5, 6]. Understanding patients’ health information-seeking behaviors and preparing information delivery policies are also important for improving the quality of life through individual healthcare and reducing national medical costs [7].

Patients with CAD need a variety of health-related information for disease management, prognosis, and establishment of long-term treatment plans, and the need for related research to support knowledge exploration and utilization to improve the self-management of heart failure patients has been emphasized [8, 9]. Health information-seeking behavior is an active self-management technique for improving one’s own health, as is seeking health-related information, and utilizing such to make decisions related to disease treatment [10]. Patients’ health information-seeking behavior helps them make correct decisions utilizing the information and influences the practice of health behaviors, such as self-management skills and improvement of treatment compliance [10, 11]. Compliance with disease prevention guidelines and recommendations is thus improved, having a positive impact on health promotion behaviors [12]. Thus, medical information-seeking behavior is closely related to patients’ health management, and understanding it is an essential element in developing strategies to improve patients’ health behavior.

Health information-seeking behavior and form are affected not only by demographic characteristics such as sex, age, and patients’ education level but also by various factors such as the presence and type of disease [13]. Today, health information delivery media is wide and diverse, including mass media (i.e., TV and radio), online media utilizing the Internet, and face-to-face contact with medical personnel, families, and acquaintances [14]. Media that patients preferentially utilize differ according to age, type of disease, education level, and information accessibility [13, 15, 16]. To successfully deliver health information, the target’s accessibility to information must be considered along with the health promotion goals [8]. Large-scale national surveys are being conducted to identify the health-information-seeking patterns of subjects and to develop and distribute differentiated health information according to demographic characteristics and disease degree [17]. To deliver health information to patients with CAD in Korea, narrowing the information gap and preparing an effective health information delivery strategy is necessary, including identifying the characteristics of subjects who utilize various information media.

Health literacy is closely related to patients’ medical information-seeking behavior [18, 19]. Health literacy refers to the ability to understand and appropriately utilize health information for disease management to approach and solve health problems [20]. Healthy adults vary in their cancer information-seeking behavior and the media they utilize depends on their level of health literacy [21]. This level in patients affects not only health information search behavior but also various health-related outcomes, such as health promotion behavior and medical utilization, recurrence, and exacerbation of diseases [6, 22]. The higher the health literacy, the higher the level of rehabilitation for stroke patients; the lower the level of health literacy for chronic stroke patients, the lower the drug compliance and health behavior practice, and the higher the frequency of emergency room utilization [22]. For this reason, the need for a national strategy, goal development, and related research to identify and improve the level of health literacy is emphasized [7, 9].

However, there is a lack of research identifying the level of health literacy in patients with CAD in Korea and confirming its relationship with health information-seeking behavior. Recently, with the development of communication technology, access to health information utilizing the Internet and mobile devices has increased, yet patients with low levels of health literacy experience difficulties searching for digital health-related information and do not properly utilize related information [13, 19, 23]. The difference between the patients’ ability to understand health information and their ability to utilize it through an information search can further increase the health gap [23]. Therefore, understanding the level of health literacy in patients with CAD is necessary and analyzing the relationship between health literacy and health information-seeking behavior.

Patients with CAD require various types of information for healthcare [9] and exploring and utilizing health information on their own is an important factor in practicing patients’ practical health behaviors [3, 4, 9]. Accordingly, this study aimed to confirm the demographic and sociological characteristics and health information-seeking patterns of patients with CAD, including their level of health literacy, and identify factors that influence health information-seeking behavior. Through this, we intend to develop effective health information delivery strategies for patients with CAD and provide basic data for preparing measures to promote health information-seeking behavior.

## Patients and methods

### Data source

This study utilized data from the 2^nd^ Korean Medical Panel Survey (KMPS, version 2.1) jointly conducted by the Korea Institute for Health and Social Affairs and the National Health Insurance Service in 2021. The survey has been conducted annually since 2008 and consists of indicators such as comprehensive academic and economic characteristics of households and household members, chronic diseases, drug use status, and health information use. It is mainly used by health policy-related institutions to establish public health and health insurance policies.

The KMPS data used the sampling framework used in the national census to secure the representativeness of the sample and applied a probability proportional two-step stratified extraction method. Analysis by complex sampling design was performed with multiple sampling, and weights were applied to reduce the potential bias of the sample. By applying weights, we wanted to reduce the risk of under- or over-reported national average and total estimates. Additionally, weights were applied in the data analysis process.

This can improve the representativeness and accuracy of the sample by correcting the error due to the population difference between the sample design time and the survey time, the uneven extraction rate, and the non-response error of non-participants [24].

**Fig 1:**
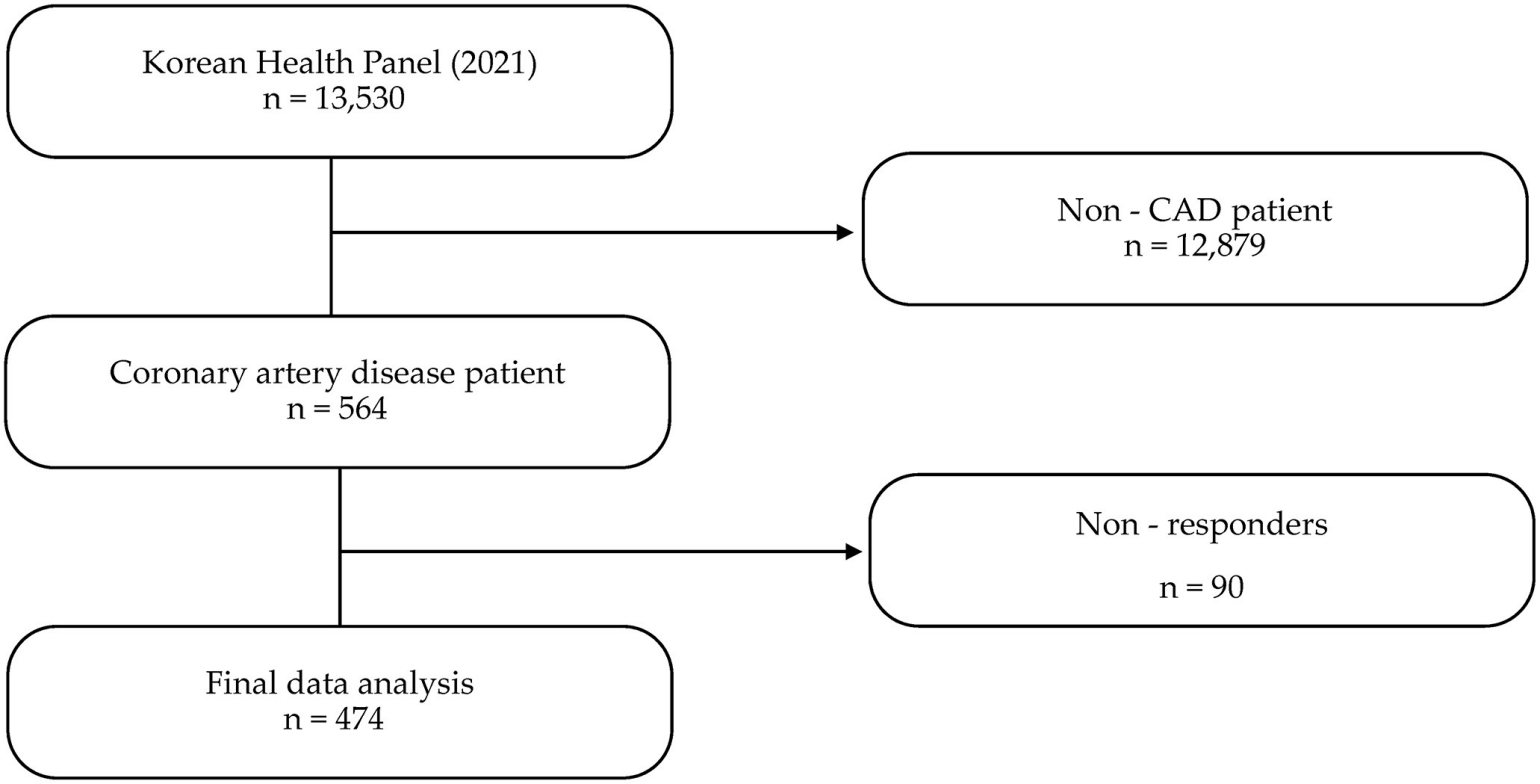
Study sampling stages.

In 2021, 13,530 people were surveyed in the second phase of the KMPS, and 564 of 12,879 adults aged 19 years or older were diagnosed with CAD by doctors. Among them, 474 people were finally analyzed, after excluding 26 who had insufficient answers for the independent variables and 64 who had insufficient answers for health literacy (Fig 1).

### Description of variables

#### Socio-demographics

The data from the KMPS utilized in this study were composite sample designs with a probability-proportional two-stage stratified colony extraction method and weighted data to secure a sample that represents the entire Korean population. Gender, age, education level, economic activity, spouse, residential type, subjective health status, and residential area were investigated as demographic characteristics of the subjects.

The age of the study participants was classified as < 60, 60–70, and > 70 years, and the level of education was categorized as elementary school graduation or less, middle school, high school, and college graduation or higher. Economic activity was categorized as “Yes” or “No” or “no”. Spouses were classified as “yes” if currently married, and “no” if divorced/separated/widowed/or never married. Living status was categorized as “alone” if living alone, and “with family” if living with two or more family members. Subjective health status was assessed utilizing a medical panel raw data questionnaire asking, “How do you think your general health is?” with answers classified as “Good”, “Moderate”, and “Poor”. Area of current residence was divided into “City” and “Rural” areas.

#### Health literacy (HLS-EU-Q16)

The KMPS data utilized in this study were collected via the health literacy measurement tool, HLS-EU-Q16. This measurement tool consists of 16 survey items developed by the World Health Organization European Regional Office. Initially, the Cronbach’s alpha value was .79, and once the tool was translated into Korean, became .86. Although the original questionnaire utilized a 4-point scale, it was calculated on a 2-point scale. For each question, “very difficult” and “difficult” were coded as 0 points and “easy” and “very easy” were coded as 1 point, for a total of 16 points. Health literacy, calculated out of a total of 16 points, is further classified into three health literacy levels: “inadequate” (0–8), “problematic” (9–12), and “sufficient” (13–16) [25, 26].

#### Health information-seeking behavior

In this study, the health information-seeking behavior of the subjects was measured utilizing the medical panel raw data questionnaire: “Have you ever looked for information about health or medical care in the past year?” A “yes” answer was classified as having health-information-seeking behaviors. The search form of subjects classified into the “Yes” group of health information-seeking behavior was determined by the question, “From which information source do you mainly get information about health or medical care?” in the medical panel raw data questionnaire, including mass media (i.e., television, radio). Information media was investigated of first choice utilization of the following: newspapers, magazines or books, face-to-face contact (healthcare workers, family, friends, colleagues, acquaintances), and online contact (government/public institution homepage, Internet portals).

#### Data analysis

Data analysis was conducted utilizing IBM SPSS version 27.0 for Windows (IBM Corp., Armonk, NY, USA). The KMPS 2021 annual data utilized in this study have a complex sampling design with a probability proportional two-stage stratified cluster sampling method. The data are weighted to a statistically corrected data bias to ensure sample accuracy and representativeness. When applying a general statistical analysis without considering this, bias in estimates occurred, and a complex sample analysis was performed considering the impossibility of generalization. Cross-analysis was utilized to identify differences in health information-seeking behavior and media searching according to the demographic and sociological characteristics and health literacy of the participants, and factors affecting health information-seeking behavior were analyzed via logistic regression analysis. In all analyses, a p value of 0.05 was considered statistically significant.

### Ethical statements

This study was conducted in accordance with the Declaration of Helsinki. The Korean Medical Panel data used in this study is statistical data designated by the government pursuant to Article 18 of the Statistics Act (Approval No. 920012). This study was exempt from ethical review and approval because only de-identified data was providesd and used in accordance with the Personal Information Protection Act and Statistics Act to prevent individuals from being inferred from survey data.

## Results

### Differences in health information-seeking behavior of CAD

This study uses data from the Korean Medical Panel, which has secured sample representation through complex sample design and weight application, for analysis, and the demographic and sociological characteristics of this study can represent the entire Korean people. As a result of the study, the proportion of CAD patients who performed health information-seeking behavior was confirmed to be 42.6%.

Differences in health information-seeking behaviors according to demographic characteristics revealed that males engaged more in health information-seeking behaviors compared to females, and the proportion of health information-seeking behaviors was higher among those under 60 years of age. Additionally, individuals with lower levels of education were less likely to engage in health information-seeking behaviors. Among CAD patients, 67.7% of those with sufficient health literacy levels had a high proportion of health information-seeking behavior, while 20.2% of those with Inadequate health literacy levels had a low proportion (χ^2^ = 32.62, *p* < 0.001) (Table 1).

**Table 1:** Differences in health information-seeking behavior of CAD (n = 474).

Characteristic	Health information–seeking behavior (Yes) n = 167 (42.6%)	Health information–seeking behavior (No) n = 307 (57.4%)	*χ* ^*2*^	p
Gender				
Male	98 (50.6)	144 (49.4)	8.00	0.005
Female	69 (33.7)	163 (66.3)		
Age (yr)				
<59	24 (60.3)	20 (39.7)	7.39	0.001
60–69	58 (53.5)	66 (46.5)		
≥ 70	85 (31.1)	221 (68.9)		
Education level				
≤ Elementary school	59 (26.8)	176 (73.2)	7.41	< 0.001
Middle school	38 (53.2)	49 (46.8)		
High school	53 (60.5)	56 (39.5)		
≥ College	16 (40.4)	26 (59.6)		
Economic activity				
Yes	80 (48.7)	124 (51.3)	3.13	0.077
No	87 (37.7)	183 (62.3)		
Presence of spouse				
Yes	120 (46.4)	195 (53.6)	1.96	0.162
No	47 (37.5)	112 (62.5)		
Living status				
Alone	34 (37.2)	86 (62.8)	1.39	0.239
With family	133 (45.3)	221 (54.7)		
Perceived health status				
Good	33 (43.5)	59 (56.5)	3.31	0.037
Moderate	81 (50.2)	116 (49.8)		
Poor	53 (33.3)	132 (66.7)		
Region				
City	114 (44.7)	195 (55.3)	1.88	0.171
Rural	53 (36.5)	112 (28.8)		
Health literacy				
Inadequate	53 (20.2)	213 (79.8)	32.62	< 0.001
Problematic	50 (60.5)	52 (39.5)		
Sufficient	64 (67.7)	42 (32.3)		

Values are presented as number (%).

Inadequate (score 0–8), problematic (score 9–12), and sufficient (score 13–16).

### Media information usage for CAD searching for health information

In this study, 167 patients with CAD who performed health information-seeking behaviors were analyzed for differences in information media utilized preferentially when searching for health information. Depending on the age group, subjects under the age of 60 often utilized online media to search for health information (χ^2^ = 2.70, p = 0.033), it was confirmed that the age group over 70 years old searched for health information through face-to-face contact and mass media rather than online (χ^2^ = 4.13, p = 0.001). Depending on the subject’s educational level, online media was preferred for high school and college graduation and above, and face-to-face contact and mass media for middle school and elementary school graduation and below (χ^2^ = 4.13, p = 0.001). Among the subjects of the study, health information was searched through online media for economic activities and face-to-face contact for non-economic activities (χ^2^ = 4.14, p = 0.013), it was confirmed that groups with good subjective health status recognition utilize online information media, and groups with poor recognition utilize face-to-face contact to search for health information (χ^2^ = 2.48, p = 0.044) (Table 2).

**Table 2:** Media information usage for CAD searching for health information (n = 167).

Characteristic	Information channel of seeking behavior			*χ* ^*2*^	p
	Mass media	Face contact	Online		
Gender					
Male	34 (28.2)	33 (31.7)	31 (40.1)	2.71	0.067
Female	25 (45.9)	29 (32.4)	15 (21.7)		
Age (yr)					
<59	6 (22.3)	6 (25.1)	12 (52.7)	2.70	0.033
60–69	19 (32.0)	19 (29.3)	20 (38.7)		
≥ 70	34 (45.7)	37 (38.7)	14 (15.6)		
Education level					
≤ Elementary school	25 (47.9)	26 (39.9)	8 (12.3)	4.13	0.001
Middle school	12 (36.8)	18 (44.9)	8 (18.3)		
High school	15 (24.5)	16 (27.3)	22 (48.3)		
≥ College	7 (35.8)	2 (5.3)	8 (58.9)		
Economic activity					
Yes	30 (32.8)	23 (22.2)	27 (45.0)	4.39	0.013
No	29 (37.1)	39 (41.9)	19 (21.1)		
Presence of spouse					
Yes	41 (31.4)	44 (31.8)	35 (36.8)	0.59	0.554
No	18 (40.6)	18 (32.2)	11 (27.2)		
Living status					
Alone	14 (43.5)	12 (28.7)	8 (27.8)	0.59	0.556
With family	45 (31.3)	50 (33.3)	38 (35.4)		
Perceived health status					
Good	8 (20.3)	12 (31.1)	13 (48.6)	2.48	0.044
Moderate	31 (37.1)	26 (25.7)	24 (37.2)		
Poor	20 (40.5)	24 (43.3)	9 (16.2)		
Region					
City	44 (37.0)	37 (27.7)	33 (35.3)	1.99	0.137
Rural	15 (27.5)	25 (46.7)	13 (25.8)		

Values are presented as number (%).

### Media information usage differences according to the level of health literacy among CAD searching for health information

**Fig 2:**
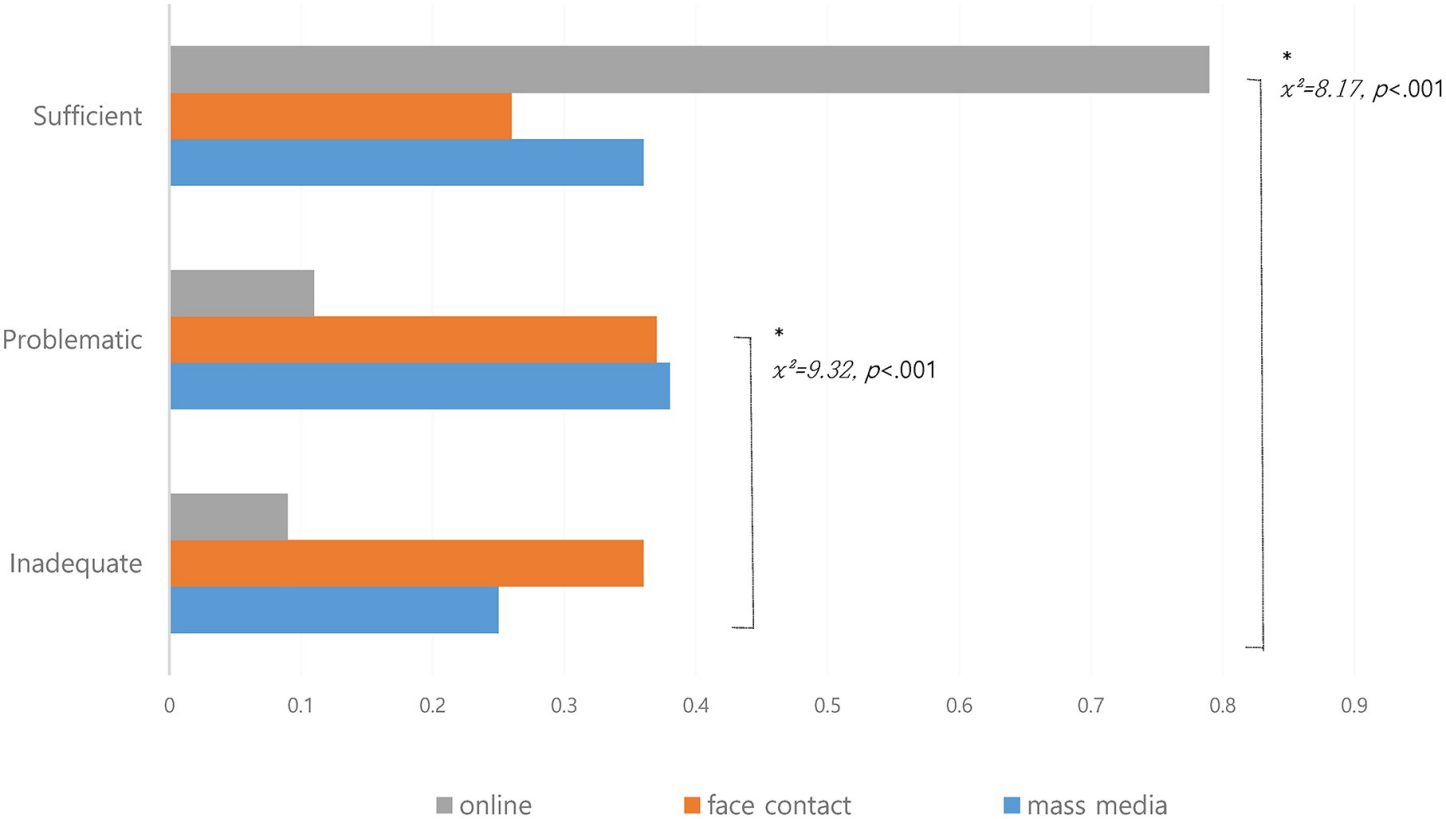
Media information usage differences according to the level of health literacy among coronary artery disease (CAD) searching for health information.

In this study, 167 CAD participants who performed health information-seeking behavior were analyzed for their preferred information media according to their level of health literacy. Those with sufficient levels of health literacy had the highest frequency of utilizing online media to search for health information (χ^2^ = 8.17, p < 0.001), and those with inadequate health literacy levels had the least frequency of utilizing online media, preferring face-to-face contact (χ^2^ = 9.32, p < 0.001) (Fig 2).

### Factors affecting the health information-seeking behavior of CAD

**Fig 3:**
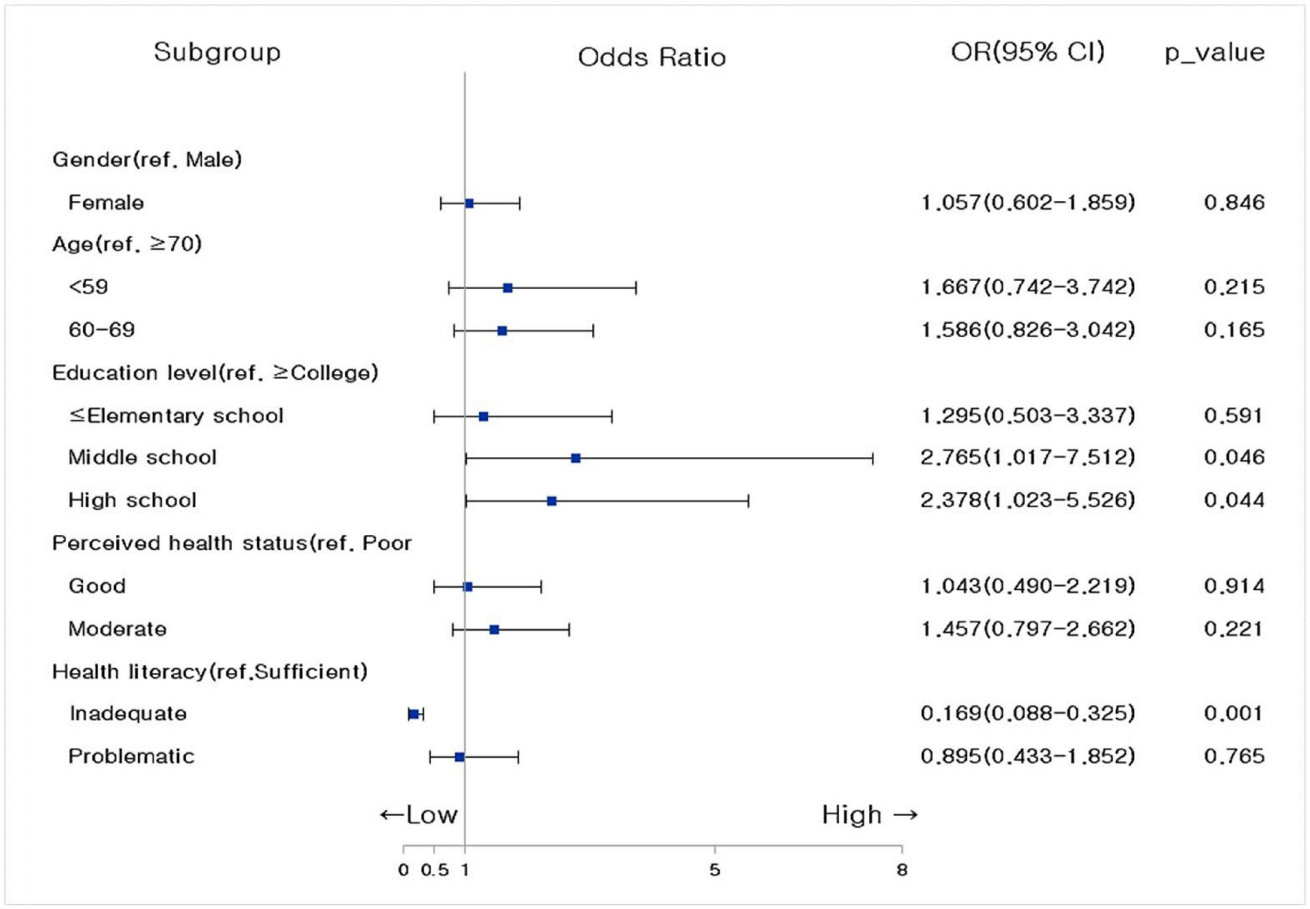
Factors affecting the health information-seeking behavior of coronary artery disease.

Logistic regression analysis was conducted to confirm the factors affecting the health information-seeking behavior of adult Korean CAD patients. Age, education level, economic activity, subjective dry state perception, and health literacy level (all showing significant differences per demographic and sociological characteristics of the participants) were utilized as independent variables. The regression model was significant (Wald F = 0.060, p < 0.001); the Cox and Snell R^2^, which represents the model’s explanatory power, was 23%, and the Nagelkerke R^2^ showed 31%. The analysis revealed that education level and health literacy were factors affecting the health information-seeking behavior of CAD patients (Fig 3).

## Discussion

This study attempted to identify differences in health information-seeking behavior according to demographic characteristics and health literacy levels of adult CAD patients, to identify factors that affect health information-seeking behavior, and to provide basic data for strategies to improve effective health information delivery and information search behavior.

### Differences in health information-seeking behavior of CAD patients

As a result of analyzing the differences in health information-seeking behavior according to the demographic characteristics of CAD patients, differences in health information search behavior were confirmed according to sex, age, education level, and subjective health status. Among the demographic and sociological characteristics of the participants, unlike women who actively implement health information search behavior compared to men [12, 18, 27], this study found that women exhibited lower levels of health information-seeking behavior.

Patients with cardiovascular disease show differences in disease perception according to sex, and emotional conditions such as individual anxiety and anger affect health information search behavior [28, 29]. Considering these points, additional studies are needed to determine the sex and emotional states of CAD patients.

Patients with low educational levels and the elderly are vulnerable groups with low access to health information because of their low interest in and frequency of use of health information [13, 15, 27]. The absence of information-seeking behavior affects patients’ negative health outcomes and quality of life and can lead to psychological and cost burdens [10, 12, 30]. Elderly patients prefer health information with sounds and images, whereas adults with lower education levels prefer health information with simplified visual images over textual forms [31, 32]. This suggests that a differentiated approach is needed to develop information media utilizing words and images that are easy to understand for low education and elderly patients with coronary artery disease.

Meanwhile, participants who lacked health literacy did not engage in health information-seeking behavior. Patients who lack health literacy do not understand the contents and instructions of medical personnel and experience difficulties in healthcare because of low access to health information [18, 19]. To manage their health, social interest and efforts are needed to identify the level of health literacy along with sociodemographic characteristics and encourage interest in utilizing health information among those who lack it.

### Media information usage for CAD patients searching for health information

To successfully deliver health information, it is important to consider accessibility and preferences of the subjects of such information [31–33]. Accordingly, this study analyzed the information media utilized as a priority by 167 participants in their search for health information. CAD participants differed in their preferred information media according to various demographic characteristics and health literacy levels.

First, it was confirmed that the age group over 70 years preferred online mass media as a health information search medium. This is the result of old age, which reflects the reality that media utilization increases due to a decrease in family members and a gradual decline in interpersonal relationships and passive utilization of health information delivered by mass media [15, 34]. Owing to the one-way nature of mass media, it is difficult to find information when needed, and there are concerns about the possibility of distorted information being delivered due to commercialization. However, mass media is required to open and manage related channels so that reliable health information can be delivered at the national level, considering this medium is the main path of health information for the elderly in Korea. Establishing a health information delivery system by actively utilizing mass media such as television and radio is a cost-effective strategy for the healthcare of elderly patients with CAD.

The utilization of online media for dispensing health information has a significant impact on improving health, regardless of education level and can play a key role in improving healthcare [30]. Owing to Korea’s high Internet penetration rate, the Internet utilization rate of people in their 60s accounts for 91.5%, and the accessibility of various information through the internet for the elderly has also increased. However, while Internet accessibility of the elderly is high, the level of information search and utilization is low at 59.8% for those in their 60s and 14.9% for those in their 70s and older [23]. Hence, it is important to prepare measures to improve the ability of the elderly to utilize online information before preparing a health information delivery policy utilizing the Internet.

The digital health sector, which has recently developed rapidly with the spread of digital technology and the Internet, has been emphasized for its active utilization in contributing to the improvement of individual healthcare by increasing access to health information and the utilization of health services [7]. However, it has been confirmed that patients with CAD who lack health literacy have a lower proportion of online media use. Patients lacking health literacy have poor access to online and digital health information utilizing the Internet, which may further increase health disparities [18, 19, 23]. Our study results confirm differences in media information utilization according to the level of health literacy. This suggests that when preparing policies for the delivery of health information, it is necessary to first evaluate and reflect on the ability of patients with CAD to understand health information.

### Factors affecting the health information-seeking behavior of CAD patients

Finally, the factor affecting the health information search behavior of CAD patients was identified as an insufficient level of health literacy. The ability to find and utilize necessary health information is paramount for CAD patients to prevent recurrence and improve healthcare through lifestyle improvements [5, 6]. Healthcare professionals must find ways to communicate effectively with people who do not seek information on their own to manage their health. Health literacy is an important means to improve people’s health, and the effectiveness of applying interventions to improve health literacy has been discussed in several studies [35]. Therefore, based on the results of this study, to activate the health information-seeking behavior of CAD patients, it is essential to assess the level of health literacy of the subjects and utilize an intervention program tailored to such. In addition, healthcare professionals should make careful efforts to help patients who lack health literacy recognize the need to seek health information and increase their utilization of it.

This study has some limitations. First, this was a secondary data analysis study utilizing KMPS data, and we were unable to utilize a specific health information search scale to collect data from a large population. Future studies should include follow-up studies utilizing valid measurement tools. Second, considering our results, we propose a longitudinal study that can investigate the causal relationship between the health information-seeking behavior of CAD patients and their health outcomes.

However, despite these limitations, this study is the first to analyze factors affecting the health information-seeking behavior of CAD patients in Korea and may be utilized to prepare healthcare policies to improve effective information delivery and health information-seeking behavior. This is significant because it provides a basis for their utilization as basic data.

## Conclusion

In this study, health information search behaviors and preferred information media were identified according to demographic characteristics and health literacy levels of CAD patients. Healthcare experts should recognize that understanding subjects should come first when providing health-related information and establishing an effective information delivery system based on such is important. An insufficient level of health literacy has been identified as a factor affecting the health information-seeking behavior of CAD patients. This suggests that to improve positive health outcomes by activating health information-seeking behavior, the application of education and intervention programs to raise the level of health literacy is necessary.
